# Hospital Meetings, &c.

**Published:** 1899-04-29

**Authors:** 


### HOSPITAL MEETINGS, &C.

### ROYAL FREE HOSPITAL.

A special meeting was held at the Mansion House on Tuesday last, the 25th instant, under the presidency of the Lord Mayor, Sir John Voce Moore, on behalf of the Buildings Improvement Fund of the Royal Free Hospital, Gray's Inn Road.

The Chairman opened the meeting with a brief *résumé* of the points of the statements issued by the committee of management and by the president of the hospital, Lord Dufferin and Ava. The appeal made is for £8,500, to enable important structural improvements to be carried out in the wards, and to provide better accommodation for the nursing staff. It is intended to build new lavatories, bathrooms, and kitchens for each ward, and separate bedrooms for most of the nurses, in place of the cubicles and large dormitories now in use. These alterations will bring the institution up to the modern standard of sanitary requirements, and are strongly recommended by the visiting committee of the Prince of Wales's Hospital Fund. Every year the demands made by the sick poor upon the resources of the hospital increase. During 1891 there were 2,151 in-patients, and 36,537 out-patients. The annual expenditure is about £13,000, the reliable income amounting to not more than £4,000. Two special claims the institution has upon public sympathy and support. It was the first general hospital in London to open its doors free to the poor without requiring letters of recommendation, and for seventy years it has adhered to this system. And it is the only general hospital in London which affords female medical students opportunities for clinical instruction necessary to enable them to qualify for degrees.

Mr. Charles Burt, the chairman of the Weekly Board, proposed the first resolution: "That this meeting, appreciating the valuable aid rendered to the sick poor of London by the Royal Free Hospital, pledges itself to use every endeavour to assist the committee of management in obtaining the necessary funds to enable them to carry out the structural improvements needed in the wards and nurses' quarters, and thereby render the institution more efficient for its beneficent work." He expressed regret at the absence of Lord Dufferin and Ava, and went on to review the history of the hospital since its inception in the winter of 1827, when William Marsden, a surgeon with a kind heart, saw a poor girl lying, after midnight, on the steps of St. Andrew's Church, Holborn, so ill that though he rendered what aid he could she died two days later. He was so impressed by the fact that there was not a single general hospital in London where such poor creatures could go without payment or recommendation, that he there and then set about establishing a medical charity, in which the only passports needed should be that the applicants were poor and sick and suffering and fit subjects for the wards. Though some of the great hospitals of London have been in existence for six or seven hundred years yet this was the first time one had been established on such principles. Briefly the speaker touched on the various points in the progress of the institution, recalling the fact that when in 1832 cholera appeared in London, though other hospitals closed their doors the Free Hospital threw theirs open and admitted over 700 cases; and in 1849 and 1854 they again received over 9,000 cases. He believed Sir Henry Burdett would bear him out in saying that they were still practically the only free hospital—that at all others either payment, ticket, letter, or something was required. But he felt theirs was the right principle. He was sure that when Saint Martin divided his cloak with the poor beggar he did not ask for a letter of recommendation. If he had he would not have seen the glorious vision he did. They had done good work, and were proud of their principles and of their work. Seven years ago they had asked for £20,000 to rebuild their front. It had cost £30,000, but he was thankful to say that every penny was paid, and having done so well they ventured to come again. This time they only asked for £8,500, though with furnishing and so forth he believed they would spend £10,000. He detailed the need for new accommodation, and the plans to be adopted to provide it. There was no doubt the work was necessary, and since the managers gave freely of their time and money they felt they were entitled to ask for help to make the best use of the institution in which they took so great an interest.

Sir Henry Burdett seconded the resolution. He recalled the circumstances of the "Stella" disaster, and said that those who lived in the midst of all the advantages of wealth and civilisation were apt to forget that any day we might be in the presence of a great disaster of another kind—a railway accident. The Royal Free was to a large extent the railway hospital, and if an accident were to occur on any of the great lines running north out of London it was to that hospital that the injured would surely be brought, and they or their friends would find their way to its doors. In so vast a city as London, with so many appeals of such various kinds, it was essential that managers should specialise their claims and drive them home, and it should be emphasised that the Royal Free Hospital, being the railway hospital, had a special claim on the vast crowds who travel by rail to and from London so continually. His second point was that the hospital was poor—and poor because it was free; those who needed its help could obtain it without inquiry and without difficulty. He had long been connected with the business life of the City, and he had never known any appeal to the wealthy and thrifty men of the City of London for a worthy object that did not meet with an adequate response. He was departing from a rule he had found himself obliged to make in appearing there on behalf of a particular charity, but he felt that if the simple facts of the case were known the £8,500 asked for would be forthcoming without difficulty and without delay. This was the first free hospital, and was still practically the only free hospital, but so little was the fact appreciated that he was bound to believe that it was little known, and that that was the reason so small a proportion of their income as 10 per cent. was derived from annual subscriptions. But this was essentially a women's hospital, the one hospital which had had the courage and independence to open its doors to women students, and so had rendered a great service to the cause of women. Surely, then, it was an institution which should be supported by women, and if it were so supported the percentage of annual subscriptions would be more like 60 per cent. than 10 per cent. This he knew from experience, and so he said to the managers: You must get ladies to work on this fund; start a ladies' committee, with a subscription of 5s., and you will soon get results. What could not the ladies do! In seven years, for the Great Northern Central Hospital, they had raised £120,000, under the guidance of Mrs. Harkness—one of the most noteworthy and encouraging events he could recall in an experience of 25 years of hospital work. He made the suggestion, and was sure that, if properly worked, the whole of the annual income needed would be raised. As to the £8,500, that should come from the City, and there would be no difficulty if once the facts were brought home. The institution was free, unsectarian, economical, essential to the district in which it was, safeguarded from abuse by thorough inquiry after admittance, enterprising, and ready to listen to any suggestion tending to add to its usefulness and efficiency. It was well administered, the attendance of the Weekly Board being the greatest in London. If these facts were brought home he had no doubt that all that was required would be forthcoming in a very short time. One word more he would say, not of censure, but as showing the need for the appeal. Though this hospital had a women's medical school, yet its accommodation for its nurses was as bad as that of any hospital in London. He had personally inspected the institution, and had been impressed with the efficiency of the nursing and with the character of the nurses, and considered they had the strongest claim to the improvements in accommodation proposed. He had the greatest pleasure in seconding the resolution.

Mr. Albert G. Sandeman and Dr. Harrington Sainsbury supported the resolution, which was carried with enthusiasm.

Mrs. Garrett Anderson, M.D., the Dean of the Women's Medical School, proposed, "That, in the opinion of this meeting, the Royal Free Hospital has special claims to public sympathy and support in that it is the only general hospital in London which has thrown open its doors to female medical students," and this was seconded by Mrs. Scharlieb, M.S., and carried.

The Secretary (Mr. Conrad Thies) announced that the donations received and promised amounted to £1,253, which, with £2,835 set apart as a nucleus for this fund by the committee at the beginning of the year, made a total of £4,123.

Mr. Edward Ford North, in proposing a vote of thanks to the Lord Mayor, appealed to the Press to make the needs of the hospital known, since, unlike some of the great hospitals, it was hidden away in a poor district, and was not constantly under the eyes of the wealthy, so that they would remember it in their distributions.

Captain Jessel, M.P., seconded, and emphasised the fact that, despite attacks upon it, the City was the place to which the poorer districts looked when they needed assistance.

The meeting then separated.

### METROPOLITAN HOSPITAL.

The record of the Metropolitan Hospital for the past twelve months has been one of constant endeavour to pull the hospital out of the hopeless state of debt in which it has been struggling for years. Energetic endeavours on the part of the committee and the secretary, coupled with a very lucky year financially, has enabled the hospital to pay its way, and, more than all, to reduce its debt of some £7,200, at which figure it stood at the beginning of 1898, to something under £5,000 at the end of 1898, and finally to about £3,500 at the present time. All this is a good record, and we welcome it the more because it proves the truth of our contention concerning this hospital in the past, viz.: that more energy in the management was all that was required to make a hospital, which is more than wanted in the neighbourhood in which it is situated, a flourishing institution doing good work at something like a reasonable cost. As Sir G. Faudel-Phillips said at the annual festival dinner which was held on Monday, the 24th instant, at the Whitehall Rooms, "Success or failure depends on the management, and this hospital may boast of good management, because they can show increased assets and decreased liabilities." We hope, however, that the management will not think that their efforts during the past year, exhausting though they may have been, are sufficient, and that they can now take a rest. They still have an uphill struggle before them, and they must continue it until they can say, "We are at last free from debt, and now we must ask the people of London to assist us to open the ninety beds which ever since the opening of the hospital have been closed for want of funds." Pluck and a dogged determination to overcome difficulties commend themselves to most people, and a continued exhibition of these qualities is bound to meet with the reward it deserves.

Mr. H. L. W. Lawson, who occupied the chair at the festival dinner, in proposing the toast "Prosperity to the Hospital," said he was glad to see that the wasteful and depressing influence of unpaid tradesmen's bills is coming to an end, and he hoped that in the future the hospital would be able to do its business on far better terms than it had been able to do it in the past. In the near future he hoped the policy of the hospital would be to aim at opening every ward which was now shut and to fill every bed that was now empty.

Mr. C. J. Thomas (the chairman of the hospital), in responding, said the hospital had had a very successful year. Nearly 1,000 in and about 30,000 out-patients had been attended. The income had amounted to £12,600, of which £5,000 was received in legacies, and the ordinary expenses had been about £8,000. In his view the fact that two members of the Visiting Committee of the Prince of Wales' Fund had paid a visit to the hospital, and that the Fund then voted a grant to the hospital of £500, showed that the report of the visitors had been a favourable one. Further than that, one of the visitors had subsequently sent the hospital a cheque for £50.

The Secretary (Mr. C. Byers) announced during the evening that contributions amounting to £2,841 had been received as the result of the dinner.

### ROYAL EYE HOSPITAL.

The annual festival dinner of the Royal Eye Hospital took place at the Hotel Metropole on Friday, 21st inst., Mr. W. M. Chinnery presiding.

General Sir William Olpherts, V.C., in responding to the toast of "Her Majesty's Forces," created great enthusiasm by coupling with it that of the American Army. In acknowledging this, Colonel Gouraud said that in his 25 years' experience of hospitality in this country he had never before heard the two armies toasted together at a public banquet; and bore eloquent testimony to the great change wrought in American public opinion by our attitude during the Spanish war.

The Chairman proposed the toast of "The Hospital." The institute was, he said, doing an immense amount of good work in relieving the sufferings of the very poor, and so enabling them to continue to earn their own living. It was situated in the heart of the most necessitous district of London, and was the only one of its kind on the south side of the river. It was known as the Model Hospital, having been designed and built for its purpose. It was unique in many respects, being arranged throughout with a view of assisting the blind inmates. Every corner was rounded, the doors were without handles, and the staircases had banisters not only at each side, but in the middle also, dividing the upgoing from the downcoming. There were no operating tables to distress the patients by their appearance; instead of that the beds were silently and easily wheeled from the ward to the operating theatre. The risk of fire was practically done away with, the place being warmed by hot water throughout, while the lifts were outside the walls of the building so that there were no shafts to create an up draught. As to the work done, there were over 17,000 cases relieved last year the number of attendances being over 48,000. Expenses were kept down to the lowest possible limit; official salaries amounted to only £88 per annum; while the medical skill at their disposal cost them but £50. They spent no money on advertising, and that was the reason, no doubt, why the hospital was less known than others—indeed, so much was this the case that he had been asked to describe its whereabouts, and to assure them that because it was near the Elephant and Castle it was not therefore near the Zoo. (Laughter.) They made a rule never to get into debt, but to avoid this they had recently been obliged to keep 14 beds idle. These, however, were now occupied. He paid a very warm tribute of praise to Professor McHardy, their senior surgeon, who, he said, was the life and soul of the institution. It was entirely due to him that the hospital had been designed and built in the extraordinary way in which it had, and specially adapted in every detail to the work to be done. And the benefit did not stop there, for such was the force of good example, that the new hospital being built for the old and famous Moorfields Institution was being largely remodelled in the same way, while the other three London eye hospitals were adopting similar plans. The collection they had made in connection with that festival was a good one and would put them on their legs for that year, but he appealed for annual subscriptions, if only of a guinea. They might not be able to help all charities, but he was certain they could not help a better or a worthier cause than this one. He concluded with a quotation from Pope:

"In Faith and Hope the world will disagree,
But all mankind's agreed in Charity."

Professor McHardy responded to the toast. Their economy of management, he said, appeared to excite great astonishment, but it was upon their absolute efficiency that they based their claim to support. He put efficiency first, and would ask for no help when their efficiency was surpassed by any other charity. He appealed for help for a kindred institution which took up the work where they left off—the Royal Normal School for the Blind at Norwood. He could not sufficiently emphasise the sense of responsibility of those who had the care of the eyes. On young students he impressed always the need for serious and careful work if they meant to be oculists. The dead did not talk; the blind could do little else, so there must be no mistakes and no carelessness where the eyes were concerned. Stock Exchange men, of whom he saw many there that night, were business men and recognised good work. Before their chairman consented to act in that capacity and to appeal to his friends on their behalf, he came down to the hospital and went through everything, and the result was a collection of £4,000, of which the chairman's list alone amounted to over £2,000. This was comparing with last year's collection of £1,404, till then their record amount. As a result they were hoping and intending to open a new ward and call it the Stock Exchange ward—not the "House" ward as was at first suggested, lest they might hurt the feelings of their poor patients, with whom that word had other associations. With the help of other City friends, Mr. Moss Myers had declared his confidence that once open that ward would not again be closed while the Stock Exchange itself remained open. On behalf of the hundreds of thousands of poor people in South London, he thanked them heartily for their help.

The Secretary (Mr. Easton) then read the list of subscriptions, comprising amounts from Messrs. Rothschild (£100), Messrs. Hambro (50 guineas), and other well-known City names, headed by the chairman himself with £200, and a most successful function was brought to a close.

